# Herbal medicine use by surgery patients in Hungary: a descriptive study

**DOI:** 10.1186/s12906-015-0890-2

**Published:** 2015-10-14

**Authors:** Sándor Árpád Soós, Norbert Jeszenői, Katalin Darvas, László Harsányi

**Affiliations:** 1st Department of Surgery, Semmelweis University, Budapest, Hungary; MTA NAP-B Molecular Neuroendocrinology Group, Institute of Physiology, Szentágothai Research Centre, Centre for Neuroscience, University of Pécs, Pécs, Hungary; Department of Genetics, Eötvös Loránd University, Budapest, Hungary; Department of Anaesthesiology and Intensive Therapy, Semmelweis University, Budapest, Hungary

**Keywords:** Herbal medicine, Perioperative care, Anaesthesia, Surgery, Drug interaction

## Abstract

**Background:**

The popularity of non-conventional treatments, especially the consumption of herbs is showing an increasing tendency all over the world. The consumption of herbal medicines might cause several complications during perioperative care.

**Methods:**

The survey was conducted at the First Department of Surgery of Semmelweis University and focused on the demographics of patients consuming herbal medicines who had undergone elective surgery between July 1^st^ 2014 and February 28^th^ 2015. A one-page questionnaire, that the patients filled in individually and anonymously, was used. The response rate was 17.3 %.

**Results:**

Out of the 390 patients who filled in the questionnaire, 7.2 % (28 patients) used herbal medicines, 3.6 % (14 patients) of them two weeks prior to their hospitalization. The other 3.6 % (14 patients) took herbal medicines sometime in the past. The majority of those who have ever consumed herbs are women (18/28), have completed secondary or tertiary education (23/28), more than half of them suffer from tumorous diseases and only a quarter of them (7/28) informed their physician about their use of herbal medication of their own accord.

**Conclusions:**

Attention must be paid to the exploration of herb consumption habits of surgery patients during the preoperative examinations in order to avoid potential side effects, complications or drug interactions.

**Electronic supplementary material:**

The online version of this article (doi:10.1186/s12906-015-0890-2) contains supplementary material, which is available to authorized users.

## Background

The popularity of Complementary and Alternative Medicine (CAM) is increasing worldwide. According to a survey, conducted in the United States, between 1990 and 1997 the application of alternative treatments rose from 33.8 to 42.1 %, the use of herbal medication was 12.1 % among the total population [1]. The usage of CAM worldwide varies broadly between 9.8 and 76 % [2]. In Korea 29 % of the total adult population uses CAM [3]. In the United Kingdom 41.4 % of the population use CAM yearly, the lifetime prevalence is 51.8 %. A rising tendency is discernible of the application of CAM among patients with tumorous disease. In the 1970s one quarter of the patients used CAM, in the 2000s, this ratio rose to 49 % [4] In Trinidad and Tobago 56.2 % of cardiac patients use alternative treatments [5]. In Hungary, one significant survey was conducted, according to which 15–20 % of the population have already used CAM [6].

So is the use of herbal medications becoming more and more widespread. According to an English study, 12.8 % of the adult population use herbal products [7]. This ratio is 5.8 % among German children and adolescents. In Kenya, 12 % of pregnant women consume herbs [8]. The perioperative period is no exception to this tendency. In Ireland 12.1 % of ambulatory surgery patients use herbal supplements [9]. The incidence of herbal medication consumption among general ambulatory surgery patients is 9.7 % according to an American survey [10]. In Nigeria 40 % of ambulatory surgery patients consume herbal medicines [11]. In the preoperative period it is very important that patients receive comprehensive care, which takes into account the use of herbal medicines as they may cause several drug interactions and complications: they may increase the possibility of blood coagulation [12] or cardiovascular problems [13], disturbances in the endocrine and electrolyte system, are potentially hepatotoxic and may prolong the effect of anaesthetic drugs [14].

CAM is typically used by a characteristic group of patients: among its users, the number of women is significantly higher and its usage is in direct proportion with the patients’ level of education and income (higher) as well as with the severity of their clinical and health condition [15]. In Canada among the CAM-using surgery patients, those with a malignant tumour used CAM more frequently [16].

In Tuscany 49.8 % of surgery patients take herbal medications or food supplements and the majority of these patients are highly educated women above the age of 48 [17]. In California 39.2 % of general surgery patients used CAM in the preoperative phase, two-thirds of them were taking herbs, the predictors include: being female, being 35–49 years old, have higher education and higher income, have sleeping or joint problems, allergies and past surgeries. It should be highlighted that 56.4 % of the patients did not inform their physician about their use of herbal medication before the operation [18].

It can be established that a significant proportion of patients do not tell their physician that they are using CAM or that they are taking herbal medication and neither do the physicians ask questions regarding the use of pharmaceutical products that do not qualify as drugs. This constitutes an important risk-factor because of the potential drug-interactions that may occur during perioperative care, therefore anaesthesiologists and other surgical professionals must be aware of the importance of this topic.

## Methods

Our primary aim was to measure what proportion of patients take some kind of herbal medication in the perioperative period.

## Study design and population

Our prospective, cross-sectional survey was conducted at the First Department of Surgery of Semmelweis University among patients who had undergone elective surgery between July 1^st^ 2014 and February 28^th^ 2015. This survey is part of a complex study about the relationship of non-conventional therapies and perioperative care. As the Department's main profile, only adults were part of the study, children were not. Otherwise there were no special inclusion criteria. Patients with emergency surgery were excluded, as the study focuses on the demographics and herbal consumption habits of the patients with elective surgery. The questionnaire was not validated. A total of 2250 patients were handled the questionnaire.

## Ethics

The study adhered to the ethical requirements of the Helsinki Declaration. Ethical approval was obtained from the Semmelweis University Regional And Institutional Committee Of Science And Research Ethics. (SE TUKEB 142/2015). Written, informed consent of the participating patients' was obtained.

## Questionnaire

A one-page questionnaire was used that patients filled in voluntarily, individually and anonymously. The first two sections of the questionnaire were concerned about socio-demographic and educational data of the participants. The third section was about the nature of the participant’s illness. The fourth concerned the prevalence of herbal medicine consumption (questions regarding the kind of herbs used were not included in the survey). The last part was concerned with the communication between the participants and their physicians about CAM. The English translation of the questionnaire is available in Additional file 1.

## Statistical analysis

We used IBM SPSS Statistics 20.0 software (SPSS Inc., Chicago IL) to process the data. We analysed the connection between herbal medication intake and age, sex, education, and the ratio of whether the patient informed the physician about herb consumption or not.

We used descriptive statistical analysis for the socio-demographic data, logistic regression and χ^2^ trial to analyse the association between the socio-demographic variables and the use or non-use of phytotherapy. Categorical responses were reported as numbers and percentages with 95 % confidence intervals. We accepted *p* < 0.05 value as a significant result.

## Results

In the examined period we received 390 appraisable questionnaires from 2250 patients who had elective surgery, the response rate was 17.3 %.

## Prevalence of herbal medicine

Twenty eight people used phytotherapy, which accounts for 7.2 % of the total number of participants: out of these 3.6 % (14 people) used it within two weeks prior to the operation. The other 3.6 % (14 people) took herbal medicine earlier than two weeks prior to the operation (at least once in their lifetime).

## Socio-demographic status

The average age of patients was 60.6 years, the youngest participant was 17, the oldest was 88 years old (SD: 13.25). The distribution of phytotherapy-using patients according to their age can be seen in Table 1. The average age of phytotherapy-using patients was 64.8 years, the youngest participant was 48, the oldest was 79 years old (SD: 9.26).

**Table 1:** The distribution of phytotherapy-using patients according to their age

Age (year)	Herbal medication		Total (*n* = 390 and %)
	Used (*n* = 28)	Not used (*n* = 362)	
10–19	0	2	2 (0.5 %)
20–29	0	5	5 (1.2 %)
30–39	0	29	29 (7.5 %)
40–49	3	46	49 (12.6 %)
50–59	6	60	66 (17 %)
60–69	9	126	135 (34.6 %)
70–79	10	80	90 (23 %)
80–89	0	14	14 (3.6 %)

Among the participants, there were 180 men (46 %) and 210 women (54 %). The distribution of patients according to their sex can be seen in Table 2. The majority of phytotherapy-using patients were women (18/28), the difference was not significant, *p* = 0.250.

**Table 2:** The distribution of herb-using patients based on their sex

Sex	Using herbal medication			Not using herbal medication (*n* = 390 and %)	Total (*n* = 390)
	In the last two weeks (*n* = 14)	Before the last two weeks (*n* = 14)	Total (*n* = 28 and %)		
Male	4	6	10 (5.6 %)	170 (94.4 %)	180
Female	10	8	18 (8.6 %)	192 (91.4 %)	210

## Disease and herbal medicine

Diseases were classified into five subgroups: inflammatory, tumorous, endocrine, other and unknown. The distribution of patients based on the type of disease can be seen in Table 3. More than half of them suffered from tumorous diseases, there difference was not significant, *p* = 0.231.

**Table 3:** The distribution of patients based on their disease

Disease	The frequency of disease (*n* = 390 and %)	Not using herbal medication (*n* = 362 and %)	Using herbal medication		
			In the last two weeks (*n* = 14)	Before the last two weeks (*n* = 14)	Total (*n* = 28 and %)
Inflammation	32 (8.2 %)	31 (7.9 %)	1	0	1 (0.3 %)
Tumours	202 (51.8 %)	187 (47.9 %)	9	6	15 (3.8 %)
Endocrine	13 (3.3 %)	12 (3.1 %)	0	1	1 (0.3 %)
Other	118 (30.3 %)	110 (28.2 %)	4	4	8 (2.1 %)
Unknown	25 (6.4 %)	22 (5.6 %)	0	3	3 (0.8 %)

## Education level and herbal medicine

Patients’ educational level can be seen in Table 4. The majority of them have completed secondary or tertiary education (23/28). The rate of using phytotherapy was significantly higher among those patients who have an academic, or at least a high school degree, compared to those who hold a lesser degree χ^2^(1) = 4.48; *p* = 0.034294; OR 2.8 (95 % CI 1.04–7.54).

**Table 4:** Phytotherapy-using patients’ qualifications

Education	The frequency of education (*n* = 390 and %)	Not using herbal medication (*n* = 362 and %)	Using herbal medication		
			In the last two weeks (*n* = 14)	Before the last two weeks (*n* = 14)	Total (*n* = 28 and %)
Elementary school	47 (12.1 %)	45 (11.5 %)	1	1	2 (0.5 %)
Vocational school	95 (24.4 %)	92 (23.6 %)	2	1	3 (0.8 %)
Secondary school	123 (31.5 %)	112 (28.7 %)	6	5	11 (2.8 %)
University, college	121 (31 %)	109 (27.9 %)	5	7	12 (3.1 %)
Post-secondary degree	4 (1 %)	4 (1 %)	0	0	0

## Communication about herbal medicine with a physician

Only 25 % of patients (7/28) informed their physician that they had been using phytotherapy two weeks before the operation or earlier (Table 5). More female patients have not informed their physician about their herb consumption, than males.

**Table 5:** The distribution of patients who reported about their use of herbal medication

Informed the physician	Using herbal medication		Sex		Average age (year)	Total (*n* = 28)
	In the last two weeks (*n* = 14)	Before the last two weeks (*n* = 14)	Male (*n* = 10)	Female (*n* = 18)		
Yes	4	3	3	4	63.3	7
Never	4	3	1	6	66.9	7
Only if asked	6	8	6	8	59.8	14

## Discussion

Our survey is the first study in Hungary that examined herbal medication consumption in the preoperative phase. The drawback of the questionnaire-format—which also accounts for the limitation of the present study—is that the accuracy of the answers depends on how honest the patients were and how well they understood the questions. However, the fact that participants were willing to give data presupposes that they fulfilled the criterion of honesty, so there is no reason to assume that they retained information on purpose, although the chances of giving a misleading answer due to inadequate understanding of some terms is higher. When seeing the expression “phytotherapy” patients do not necessarily think of the herbal teas and herb containing food supplements they take arbitrarily, without consulting their doctor. 2004/24/EC European Council directive provided definitions of herbal medicine products, substances and preparations. The latter is obtained by physicochemical treatments of herbal substances (e.g. distillation, extraction, fermentation, purification, etc.). Despite the definitions, patients might not see such herbal preparations (tablets, pills, etc.) as herbs. This may lead to the over or underestimation of our results.

According to the literature, the ratio of herbal consuming patient varies between 4.8 and 32 %, and the most frequently used herbs were Echinacea, Gingko Biloba, St. John’s Wort, garlic and ginseng [19–21]. Our results from the survey show that the consumption of herbal medication among surgery patients are lower than the average rate as we would have expected based on the corresponding literature. This is true both in the preoperative period (3.6 %) and in terms of the whole population (7.2 %).

Our next task involved the demographic description of herbal medication consuming patients and identified the predictors of herbal medication consumption. Based on our results we can establish that more than the half of those patients who consume herbal medication suffer from tumorous diseases, two-thirds of them were women, even though these factors did not constitute a significant difference. Higher education proved to be a significant factor in our survey: the rate of herbal medication usage was higher among patients who completed secondary or tertiary education.

In terms of patients’ safety in perioperative care it is a crucial point that patients must inform their physician about all the substances they take, including substances that do not qualify as drugs, and it is the physician’s responsibility to ask explicit questions about this topic when examining the patients before the operation.

We must not ignore the fact that several studies question the role that herbs might play in the development of perioperative complications [22, 23]. According to the Second ASRA (American Society of Regional Anaesthesia and Pain Medicine) Consensus Conference on Neuraxial Anaesthesia and Antithrombotic Therapy the incidence rate of epidural or spinal hematoma in neuroaxial anaesthesia is not higher among those patients who consume herbal medicines [24]. In 2000 the ASA (American Society of Anaesthesiology) published a recommendation for patients to call attention to the importance of informing their physician about all the herbs they are taking before the anaesthesia because these substances may cause several drug interactions during the treatment [25]. In line with the ASA’s recommendation several studies have suggested that patients should stop taking herbal medications two weeks before the surgery [26–29].

The survey conducted in the UK has come to the conclusion that anaesthesiologists should pay more attention to getting more information about patients use of herbal medicines in preoperative care [30]. Unfortunately, our results revealed that a mere 25 % of patients informed their physician about their use of herbs without being asked to, regardless of whether they had been taking these medications during or before the perioperative period.

## Conclusions

The present study is a pioneer survey in examining the herbal medication consumption habits of Hungarian patients. Albeit this is not a multicentral study, despite the low response ratio, based on our results it can be established that the majority of our herbal medication consuming surgery patients are highly educated women and the prevalence of herbal consumption in the preoperative period is 3.6 %.

To get a more detailed picture about this topic it would be necessary to carry out a survey which would examine the prevalence rate in the consumption of the individual species of herbs separately. Considering the potential complications of herb consumption this is an urgent question from the point of view of patients’ safety.

It can be recommended that anaesthesiologists and surgeons should devote more time to discussing this question with their patients and draw the patients attention to the importance of providing their physician with sufficient information in order to ensure the safety of the treatment.

## Additional file

Additional file 1:
**The English translation of the questionnaire handled to the patients.** (DOCX 13 kb)

## Abbreviations

ASA: American Society of Anaesthesiology
ASRA: American Society of Regional Anaesthesia and Pain Medicine
CAM: Complementary and Alternative Medicine
CI: Confidence interval
OR: Odds ratio
