# Supplementary material for: Herbal medicine use by surgery patients in Hungary: a descriptive study
Source: BMC Complement Altern Med. 2015 Oct 14;15:358. doi: 10.1186/s12906-015-0890-2 (PMC4604727; doi:10.1186/s12906-015-0890-2)
Supplement: Additional file 1: — The English translation of the questionnaire handled to the patients. (DOCX 13 kb) [file 12906_2015_890_MOESM1_ESM.docx]

Dear Patient,

please, help our research, conducted in the Doctoral School of Clinical Medicine of the Semmelweis University, by completing the following questionnaire.

The questions of this questionnaire concern the consumption of herbal remedies. We would like to assess your opininon in about these methods, do you require such treatments in health care, have you used such methods recently, especially in the perioperative period.

The completing of the questionnaire is voluntary and anonymous, your choice about completing the questionnaire or not, does not influence your treatment. If you decide to complete the questionnaire, please be aware, that your positive or negative opinions are both very important for us. There are no right or wrong answers. Please do not miss a single issue.

If you complete the questionnaire, please answer all the questions!

Please return the completed questionnaires to the nurse room!

We are grateful for your contribution!

Yours faithfully

Sándor Soós MD

Semmelweis University

1st Department of Surgery

**Age:** ………

**Sex:**

Male ⁯

Female ⁯

**Education:**

Elementary school ⁯

Vocational school ⁯

Secondary school ⁯

University, college ⁯

Academic degree ⁯

**Your disease is..:**

Inflammatory ⁯

Tumourous ⁯

Endocrine ⁯

Other ⁯

Unknown ⁯

**Have you used herbal medication?**

In the last two weeks ⁯

Before the last two weeks ⁯

Never ⁯

**Have you informed your physician about using herbal medication?**

Yes ⁯

Never ⁯

Only if the physician asked ⁯
